# Stable isotope signatures of large herbivore foraging habitats across Europe

**DOI:** 10.1371/journal.pone.0190723

**Published:** 2018-01-02

**Authors:** Emilia Hofman-Kamińska, Hervé Bocherens, Tomasz Borowik, Dorothée G. Drucker, Rafał Kowalczyk

**Affiliations:** 1 Mammal Research Institute, Polish Academy of Sciences, Białowieża, Poland; 2 Fachbereich Geowissenschaften, Forschungsbereich Paläobiologie, Universität Tübingen, Tübingen, Germany; 3 Senckenberg Center for Human Evolution and Palaeoecology (HEP), Universität Tübingen, Tübingen, Germany; Michigan Technological University, UNITED STATES

## Abstract

We investigated how do environmental and climatic factors, but also management, affect the carbon (δ^13^C) and nitrogen (δ^15^N) stable isotope composition in bone collagen of the two largest contemporary herbivores: European bison (*Bison bonasus*) and moose (*Alces alces*) across Europe. We also analysed how different scenarios of population recovery- reintroduction in bison and natural recovery in moose influenced feeding habitats and diet of these two species and compared isotopic signatures of modern populations of bison and moose (living in human-altered landscapes) with those occurring in early Holocene. We found that δ^13^C of modern bison and moose decreased with increasing forest cover. Decreasing forest cover, increasing mean annual temperature and feeding on farm crops caused an increase in δ^15^N in bison, while no factor significantly affected δ^15^N in moose. We showed significant differences in δ^13^C and δ^15^N among modern bison populations, in contrast to moose populations. Variation in both isotopes in bison resulted from inter-population differences, while in moose it was mainly an effect of intra-population variation. Almost all modern bison populations differed in δ^13^C and δ^15^N from early Holocene bison. Such differences were not observed in moose. It indicates refugee status of European bison. Our results yielded evidence that habitat structure, management and a different history of population recovery have a strong influence on foraging behaviour of large herbivores reflected in stable isotope signatures. Influence of forest structure on carbon isotope signatures of studied herbivores supports the “canopy effect” hypothesis.

## Introduction

Large mammalian herbivores experienced the most dramatic change in species composition and distribution over the last several thousand years [1, 2]. Most species went extinct in the late Pleistocene, and the surviving species experienced a dramatic decline in numbers and contraction in their range [3–6]. As a consequence, in many ecosystems in Europe their functional role in shaping the structure, composition and dynamics of plant communities has been strongly reduced, or has completely disappeared [7]. However, despite limited ranges and low densities, naturally recovering or reintroduced populations of large ungulates still play an increasing role in shaping habitats by reducing vegetation density, increasing their heterogeneity and tree recruitment [8, 9]. As a result, the recent European and worldwide trend toward rewilding landscapes has been highly promoted [10, 11].

Stable isotopes, which are transferred from plants to animal bone collagen, are increasingly used to track and reconstruct foraging habitats and diets of herbivores [12–14]. It allows for comparative analysis of modern and historically living individuals of the same species and to track their ecological plasticity and the reaction when confronted to changes in climate and vegetation [15, 16]. It also allows investigate the influence of management on spatial and foraging behaviour.

In European temperate and boreal forests, mainly C_3_ photosynthetic pathways plants occur. Therefore detection of particular plant species eaten by herbivores is not possible using δ^13^C concentration in their bone collagen, as it is the case in Africa, where differences in δ^13^C between C_3_ and C_4_ plants are used [17]. It was found, however, that plants growing under the canopy of densely forested environments are depleted in δ^13^C in comparison to plants grown in open conditions [18, 19]. It is known that bones of animals are remodeling during their whole lives with new atoms of nitrogen and carbon incorporated from the diet [20, 21]. Forest cover influences the δ^13^C of plants and consequently herbivores collagen and the value of δ^13^C of collagen below -22‰ are characteristic for the feeding in highly forested areas. Therefore, we can expect that δ^13^C of bone collagen will primarily reflect selection of foraging habitats (forest *versus* open habitats) by large herbivores.

Grazing in comparison to browsing should be reflected in higher nitrogen isotope composition, because the δ^15^N values are higher in graminoids, herbs and forbs than in trees and shrubs [12, 22]. In Europe, native C_3_ plants are expected to have δ^13^C values: from -35‰ to -20‰ [23] and cultivated maize *Zea mays* as C_4_ plant values on average -9.6‰ [24]. Therefore, herbivore populations intensively fed with maize or depredating this crop should have much higher δ^13^C than populations feeding on natural sources (C_3_ plants) only. The use of agricultural crops should also increase concentration of δ^15^N in herbivore collagen [25].

European bison (*Bison bonasus*, Linnaeus, 1758) and moose (*Alces alces*, Linnaeus, 1758) are among the few species of large herbivores in Europe that survived the climatic and environmental changes during the Pleistocene-Holocene transition [5, 26]. Today, free-ranging populations of these two large terrestrial herbivores might be found only in a restricted number of European countries, essentially in the central and eastern part of the continent. The history of European bison and moose during the Holocene is characterised by the gradual shrinkage of their ranges in Europe and their disappearance from large areas [3, 4]. After extirpation in the wild at the beginning of 20^th^ century, the European bison was reintroduced mostly to forested areas of eastern Europe and today occurs in the wild in over 35 isolated locations [27]. The number of bison has been increasing, and in total, the free-ranging population is estimated at over 4000 individuals. Bison are actively confined to the forest habitats by supplementary feeding, relocations and culling in the majority of free-ranging populations, to reduce migrations out of the forest and mitigate conflicts with farmers [28]. In contrast, the population of moose is much higher, with a total number of individuals in Scandinavia, the east Baltic countries, and European Russia estimated at 720,000, and across the majority of their distribution, moose are subject to hunting [29, 30].

Diet of European bison is dominated by grasses, sedges and herbs [31], and the species is recognised as a mixed feeder [32, 33] or even a grazer [34]. Moose is generally considered as a browser [35, 36]. Its diet includes various woody materials, such as shoots, bark, foliage (leaf stripping) and fallen leaves, which exhibit different δ^13^C and δ^15^N values [37]. It also includes a diversity of plant groups such as trees, shrubs, aquatic vegetation, graminoids and forbs [35, 38, 39], which display δ^15^N values that are highly variable [22].

In response to climate change, the early Holocene in central and eastern Europe was characterized by strong habitat change related to expansion of mixed and deciduous forest replacing tundra-steppe [40]. Early phases of forest succession and still quite opened environment with negligible human impact yet, although influencing the foraging conditions for large ungulates, allowed for selection of optimal habitats and resources [16]. The range of δ^13^C between -20.7 and -19.9‰ indicating relatively open landscape was registered in the European bison collagen living in the Early Holocene, which was in accordance with vegetational reconstructions in Denmark and northern Germany during that time [16]. Modern Europe although relatively opened, offers limited space for large ungulates, that are confined mainly to forest habitats that are very often fragmented, since the open habitats are occupied by farmland and human settlements [30].

Identification of dietary and habitat preferences is crucial for conservation and reintroduction programs. Most frequently, contemporary distribution and the pattern of habitat use of large herbivores, does not necessarily reflect natural selection for optimal foraging habitats or resources, but are strongly modified by historical context or human activity, including limited access to optimal habitats due to their disappearance or fragmentation, or the active confinement of animals in sub-optimal or marginal habitats [28, 41]. Active management of a species in suboptimal habitat due to the inaccurate or poorly informed perceptions of its historical distribution and ecology is extremely problematic and ineffective [42]. To overcome these biases, ecological information derived from ancient populations of herbivores not significantly affected by human influence may help in reintroductions and conservation management.

In this paper we aimed to 1) investigate how different factors including environmental conditions (e.g. forest cover) and climate influence the isotopic pattern of both carbon and nitrogen isotopes in bone collagen of two large herbivores across their geographical range; 2) compare feeding habitats and diet of two species that underwent different scenarios of population recovery; 3) compare the δ^13^C and δ^15^N values for modern bison and moose occurring in a human-altered landscape, with those living in pre-Neolithic times (early Holocene) before intensive agricultural activities and livestock breeding.

We expected that similarly as in plants [43, 44], δ^13^C will decrease and δ^15^N will increase with increasing temperature [45]. We predicted a positive correlation between δ^13^C of bone collagen and mean annual precipitation and between δ^13^C and altitude. Feeding at higher altitudes supposed to result in lower δ^15^N of collagen than at lower altitudes [46]. Such a complex set of factors was not previously associated to isotopic studies on European ungulates. We also expected that supplementary feeding (including maize being a C_4_ plant type) widely used in European bison management will affect the δ^13^C and δ^15^N stable isotopic composition of their bone collagen. In respect to different scenarios of population recovery in both species, we hypothesized that bison isotopic patterns will reflect the structure of habitats to which they were introduced and actively confined to those habitats, while moose will show more natural selection in effect of natural recolonization after population decline and range contraction. Because European bison and moose are two species differing in feeding strategies (mixed feeder vs. browser) and social structure (gregarious bison vs. less social moose) [27, 39], we expect differences in intra- and inter- population variation in stable isotope concentration in these two species.

Because the bison is a refugee species adapted to open and mixed habitats, that no longer have access to preferred habitats and was re-introduced to non-optimal forested habitats [28], we hypothesize that δ^13^C and δ^15^N of modern populations of bison will be different than those of prehistoric ones. In contrast, we did not expect differences between modern and Early Holocene moose as the species naturally recolonized areas of Europe previously occupied by this species and selects more optimal habitats.

## Materials and methods

### Bone origin and sample collection

**Fig 1:**
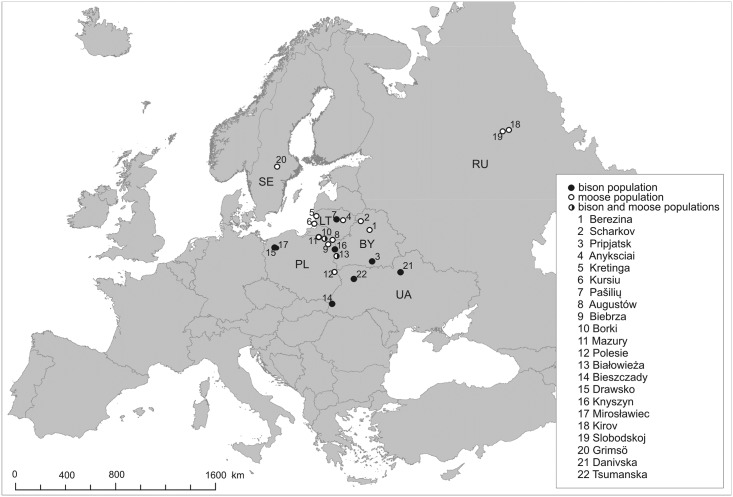
Distribution of European bison (*Bison bonasus*) and moose (*Alces alces*) populations used in the study. Projection of the map is ETRS89 / ETRS-LAEA.

The study was based on museum specimens. No animals were killed specifically for this study. No permits were required for the described study. Bone material of bison and moose was collected from zoological, museum and private collections. Samples of compact bone (0.5–1 g) were taken from the skull or long bones. Bison material originated from 1990–2013, and moose material originated from 1973–2013. Analysis of carbon and nitrogen stable isotopes in collagen was based on 79 bone samples of bison from 10 localities in 4 countries (Belarus—BY, Lithuania—LT, Poland—PL and Ukraine—UA) and 37 samples of moose from 14 localities from 5 countries (Belarus, Lithuania, Poland, Russia—RU and Sweden—SE) in Europe. The localities ranged between 15.55–50.79E, and 49.04–59.67N (Fig 1, S1 Table).

To compare present herbivore samples with pre-Neolithic specimens, we used published data for early Holocene (12,000–10,000 cal. yrs BP) bison (N = 5) and moose (N = 11) excavated in northern Europe [16, 47].

### Collagen extraction and stable isotope analysis

Collagen extracted from bones served as the basis for elemental and isotopic measurements of δ^13^C and δ^15^N. In order to remove lipids from fresh bones small pieces of compact bone were soaked in a chloroform- methanol (1:2) solution in an ultrasound bath and then rinsed with acetone and distilled water. Bones were then crushed to a powder with a mortar and sieved to obtain grain size no larger than 0.7 mm. Collagen was purified according to a protocol as in Bocherens et al (1997) [48].

The elemental and isotopic measurements were performed at the Department of Geosciences at the University of Tübingen (Germany) using an elemental analyzer NC 2500 connected to a Thermo Quest Delta+XL mass spectrometer. The isotopic ratios were expressed using the “δ” (standard delta notation) value as follows:

$$\delta^{13}C=[\frac{{{(}^{13}C{/}^{12}C)}_{sample}}{{{(}^{13}C{/}^{12}C)}_{reference}}-1]*1000\left(‰\right)$$ (1)

and

$$\delta^{15}N=[\frac{{{(}^{15}N{/}^{14}N)}_{sample}}{{{(}^{15}N{/}^{14}N)}_{reference}}-1]*1000\left(‰\right)$$ (2)

with the internationally defined standards V-PDB for δ^13^C values and atmospheric nitrogen (AIR) for δ^15^N values. Samples of collagen were calibrated to δ^13^C values of USGS24 (δ^13^C = -16.05‰) and to δ^15^N values of IAEA 305A (δ^15^N = 39.80‰). The reproducibility was ±0.1‰ for δ^13^C and ±0.2‰ for δ^15^N measurements based on multiple analysis of purified collagen from modern bones.

Even if we expect collagen from modern bones to be well preserved, we cannot totally exclude some damaging effects during the preparation of the material, so the reliability of the isotopic signatures of the collagen extracts was addressed using their chemical composition. Only high quality extracts with %C, %N, and C/N similar to those of collagen extracted from fresh bone were used for isotopic measurements. Therefore, we checked that all collagen extracts had atomic C/N ratios with 2.9 ≤ C/N ≤ 3.6 [49] and %C > 8% and %N > 3% [50].

Because bone samples originated from individuals that died in different years over a period of 40 years (1973–2013) and δ^13^C composition of global atmospheric CO_2_ vary over time [51], the δ^13^C_coll_ values measured in the collagen of modern bison and moose have been corrected for the shift in δ^13^C values because of anthropogenic CO_2_ emissions to values δ^13^C_cor_ (S1 Table) according to following formula δ^13^C_atm_ = −6.429–0.0060 exp [0.0217(t—1740)] [52], where *t* is the year of the animal’s death (S1 Table). Because the δ^13^C value for pre-industrial atmospheric CO_2_ is similar to that of the Holocene [53] δ^13^C_cor_ can be directly compared to those of Holocene bison and moose from previous studies.

### Spatial and climatic data

For each of the sampled modern populations we assigned the following parameters: (1) percentage of forest cover, (2) the mean annual temperature, (3) the mean annual precipitation and (4) altitude. Additionally, for bison populations we used data on (5) farm crops damage and (6) occurrence of C_4_ plant species (maize) in the diet of the particular population.

We used telemetric data of all previously studied and collared animals in each population to determine the range of bison and moose populations to assess proportion of forest cover within each population range. Equivalent to the population range size was the minimum convex polygon (MCP) (outlined by the peripheral locations for areas used by animals in each population) of all individually tracked animals in each local population. This was possible for bison populations from Białowieża Forest, Western Pomerania and Knyszyn Forest as well as for populations of moose from the Biebrza Marshes and Polesie Lubelskie in Poland. We also used published data on bison population ranges in Bieszczady (PL) [54, 55], Danivska (UA), Tsumanska (UA) (P. Khoyetskyy, personal communication, National Forestry University of Ukraine, Lviv), Pripjatsk (BY) [56] and Pašilių Forest (LT) [57] and moose population ranges in Grimsö (SE) [58]. They covered from 167 km^2^ in Drawsko to 1300 km^2^ in Polesie, 467 km^2^, on average. For populations with unknown exact range size, we created a buffer of 12.6 km (~500 km^2^) around a central point of the range designated by the locations of the specimens used for analysis. Such a buffer size corresponds to the average range area of the radio-tracked bison and moose populations [27, 59]. The percentage of forest cover for MCP’s or circular buffer areas were estimated on the basis of available raster data using ArcMap in ArcGIS 9.3.1 Desktop. We used CORINE Land Cover 2006 (CLC2006) raster data from a single static layer for 2006 year to obtain results with identical resolution (http://land.copernicus.eu/pan-european/corine-land-cover) for populations in Poland, Lithuania and Sweden. Because CORINE Land Cover maps were not available for Russia, Belarus and Ukraine, forest cover estimations for these areas were made using Global Forest Change maps 2000–2013 (Hansen GFC2013) [60]. To check if the forest cover has change between 2006 and year of the sample collection, whenever possible, we compared forest cover for analyzed layer and available layer the closest to the year of sample collection. In the majority of the study areas, only fragments of the forests has transformed from one forest category to another. The calculated forest cover changes were not significant and varied from 0 to 0.4%. Because high resolution maps are not available for some countries for period before 1990s, to make sure that no significant changes in the forest cover were registered, we additionally followed through forest cover visualization from LANDSAT scans on Google Earth Engine Timelapse (https://earthengine.google.com/timelapse).

Climatic data (mean annual temperature and mean annual precipitation for 1970–2000) and altitude for each population were obtained using WorldClim Version 2 data sets—interpolations of observed data based on principles of spatial autocorrelation and dependence, representative of 1970–2000—with 30 arc-seconds (~1 km) spatial resolution. Extraction of values from WorldClim data for studied localities was performed in ArcGIS 9.3.1 using spatial analysis tools. Because the similar climatic changes were registered in all countries of Central and Eastern Europe over the last few decades [61] we took an average annual temperature and precipitation for 1970–2000. This is justified because bison and moose are long-living species and the isotopic signatures of bones of such a big animals reflect the average of their whole life-time.

We obtained data on farm crop damage by bison populations, and data on the presence of C_4_ plants (maize) among cultivated crops in the bison’s range as well as their presence in bison supplementary fodder, from the literature or from interviews with local managers (S1 Table).

### Statistical analyses

We used δ^13^C_cor_ and δ^15^N values as proxies of habitat and dietary selection by European bison and moose. To model which factors affected the abundance of carbon and nitrogen isotopes, we used the following set of independent variables: locality, percentage of forest cover, mean annual temperature, mean annual precipitation, altitude, the presence/absence of crop damages, as well as the presence of C_4_ plants. We ran separate models for each species. We checked collinearity between the potential explanatory variables with Pearson’s correlation coefficients. Because of the high correlation between some predictor variables, only the uncorrelated predictor variables (R < 0.4) were included in the models. From the whole set of possible predictor variables, for bison, we applied the following continuous variables: percentage of forest cover, mean annual precipitation, and mean annual temperature. In addition we added to the model the presence/absence of crop damages as a categorical variable. For moose, we used: percentage of forest cover, mean annual precipitation, and mean annual temperature. We then ran multiple linear regression models with isotope abundance as the response variable. The Akaike Information Criterion (AIC) with the second-order correction for a small sample size (AICc) was used for model ranking [62]. The model with the lowest AICc was selected as the best model. The test for normality and homoscedasticity in distribution of final model residuals was conducted based on visual inspection of quantile–quantile distribution plot and model residuals against fitted values (estimated responses) plot. As the assumptions of normality and homoscedasticity of model residuals were met, we did not transform either response or independent variables.

One-way analysis of variance (one-way ANOVA) was performed separately for δ^13^C_cor_ and for δ^15^N in bison and moose populations. We took into account only populations with four or more analysed specimens. First, a Shapiro-Wilk normality test was applied for each population. Second, a Brown-Forsythe *F*-test (homoscedasticity) was performed to check the homogeneity of variances among different bison or moose populations. When these two assumptions were upheld, ANOVA was applied for bison and for moose. If significant differences were found in the analysis of variance, Tukey’s HSD (Honestly Significant Difference) test for unequal n (Spjøtvoll/Stoline tests) was used to assess whether and which bison/ moose populations differed.

To test the difference between isotope compositions of modern and early Holocene populations of bison and moose, we applied a Student′s *t*-test for normally distributed variables (δ^15^N in bison and moose) and a non-parametric Mann-Whitney *U*-test for non-normally distributed variables (δ^13^C_cor_ in bison). For comparisons, we used only populations where isotope abundances were measured for at least 4 individuals.

Analysis of variance, statistical tests and figures were performed in Statistica (version 9.1). Multiple regression models were completed in R (version 3.1.1).

## Results

### Species variability

**Fig 2:**
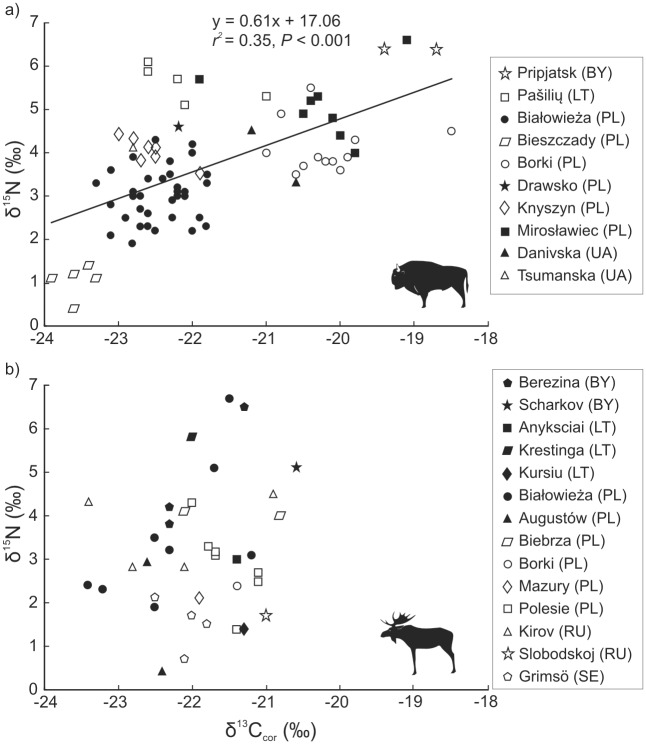
Relationship between compositions of carbon δ^13^C_cor_ and nitrogen δ^15^N isotopes in bone collagen of (a) European bison and (b) moose from different populations.

The mean values of δ^13^C_cor_ and δ^15^N did not differ between bison and moose: δ^13^C_cor_ = -21.8±1.2‰ and δ^13^C_cor_ = -21.9±1.5‰ respectively (*P* = 0.7), and δ^15^N = 3.7±1.3‰ and δ^15^N = 3.1±0.7‰ respectively (*P* = 0.06) (S1 Table). However, the range of δ^13^C_cor_ values in bison was greater than in moose (Fig 2). A significant positive correlation was found between δ^13^C_cor_ and δ^15^N in bison (*r*^*2*^ = 0.35, *P* < 0.001) (Fig 2a), but not in moose (*P* = 0.28) (Fig 2b).

**Fig 3:**
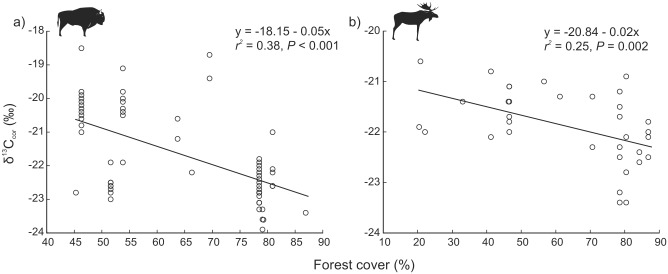
Influence of forest cover on carbon isotope compositions δ^13^C_cor_ in populations of (a) European bison and (b) moose.

**Fig 4:**
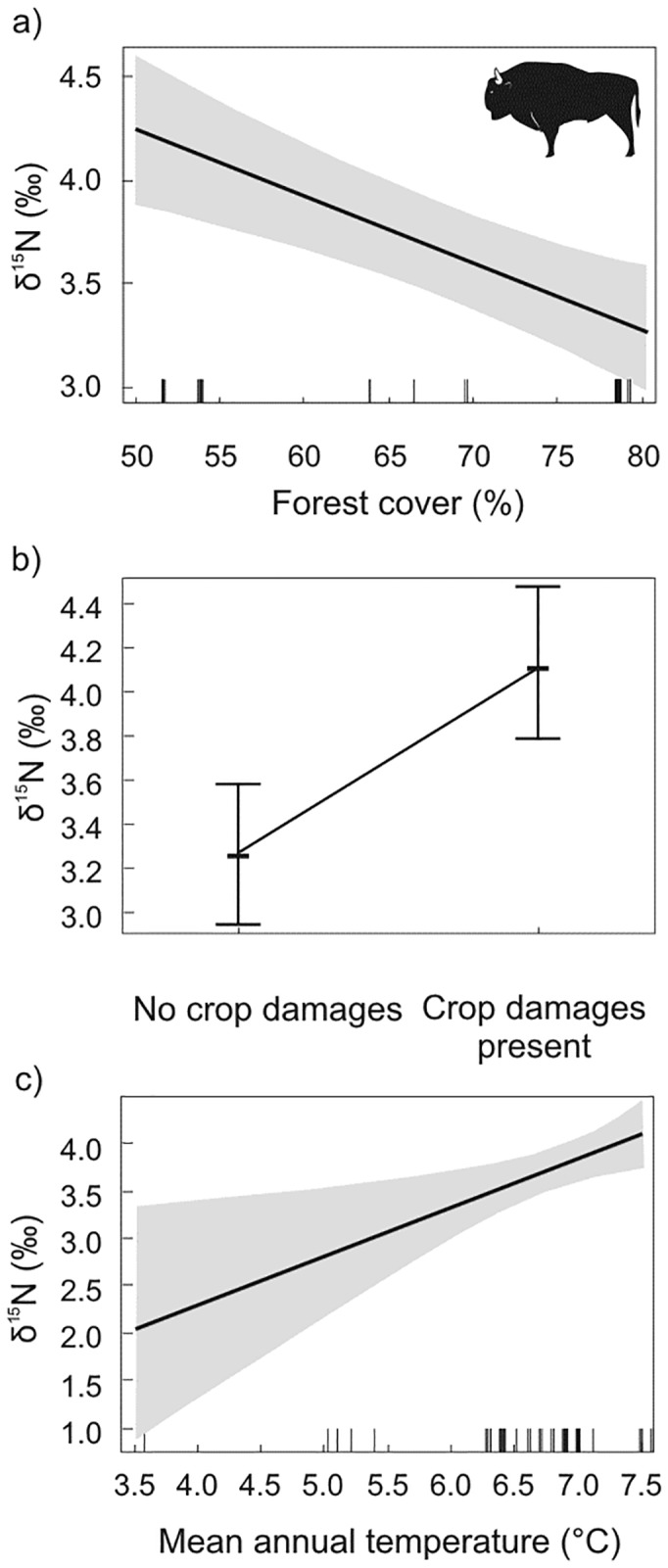
Influence of (a) forest cover, (b) presence of crop damage and (c) annual temperature on nitrogen δ^15^N isotope compositions in European bison based on estimates from multiple regression model.

The variation in carbon δ^13^C_cor_ of bison collagen was best explained by the single effect of forest cover only (Table 1). The δ^13^C_cor_ values showed a decreasing trend with increasing forest cover (*P* < 0.001) (Table 2, Fig 3a). The abundance of δ^15^N was best described by the joined effects of forest cover, crop damage and mean annual temperature (Table 1). Decreasing forest cover, increasing mean annual temperature and occurrence of damage to crops caused a significant increase in δ^15^N (Table 2, Fig 4a, 4b and 4c). Among these factors, forest cover had the highest level of significance (*P* < 0.001).

**Table 1:** Multiple regression model selection (based on the AICc criteria) to investigate the effect of different factors (see Materials and methods) on carbon (δ^13^C_cor_) and nitrogen (δ^15^N) stable isotope compositions in bone collagen of European bison and moose from different European populations.

Parameters included	N	df	AIC_c_	ΔAIC_c_	ω*i*
**Bison δ**^**13**^**C**_**cor**_	79				
Forest cover		3	227.4	0	0.550
Annual precipitation + Forest cover		4	229.4	2.04	0.199
Crop damage + Forest cover		4	229.6	2.19	0.184
**Moose δ**^**13**^**C**_**cor**_	37				
Forest cover		3	73.9	0	0.582
Annual temperature + Forest cover		4	76.2	2.32	0.182
Annual precipitation + Forest cover		4	76.4	2.48	0.168
**Bison δ**^**15**^**N**	79				
Annual temperature + Crop damage + Forest cover		5	232.0	0	0.914
Crop damage + Forest cover		4	236.9	4.92	0.078

First models on the list for δ^13^C_cor_ and δ^15^N representing the highest parsimony (the lowest AICc scores) were selected as the best models. Parameter estimates of the best models are presented in Table 2.

df—number of estimated parameters; AICc–Akaike’s information criterion with a second order correction for small sample sizes; ΔAICc–difference in AICc between a given model and the most parsimonious model; ωi–weight of the model.

**Table 2:** Parameter estimates for the best multiple regression models (Table 1), describing the effects of different factors on the carbon (δ^13^C_cor_) and nitrogen (δ^15^N) stable isotope compositions in bone collagen of European bison (N = 79) and moose (N = 37) from European different populations.

Variables	Estimate	SE	*t* - value	*P* - value
**Bison δ**^**13**^**C**_**cor**_				
Forest cover	-0.055	0.008	-6.69	**< 0.001**
**Bison δ**^**15**^**N**				
Annual temperature	0.50	0.19	2.68	**0.009**
Crop damage (present)	0.87	0.24	3.60	**0.001**
Forest cover	-0.03	0.01	-3.92	**< 0.001**
**Moose δ**^**13**^**C**_**cor**_				
Forest cover	-0.018	0.005	-3.280	**0.003**

In moose, the variation in δ^13^C_cor_ values was best explained by a model with forest cover as a single explanatory variable (Table 1). Increasing forest cover caused a significant decrease in δ^13^C_cor_ (*P* < 0.003) (Table 2, Fig 3b). No factors affected δ^15^N abundance in moose.

### Inter-population variability

In bison, the analysis of variance showed high variation in δ^13^C_cor_ and δ^15^N between populations (Fig 2a, Table 3). ANOVA post-hoc tests showed significant differences in δ^13^C_cor_ for eleven out of fifteen pairs of bison populations and for thirteen out of fifteen pairs in δ^15^N (Table 4). Bison populations from Mirosławiec (PL) and Borki (PL) recorded the highest mean δ^13^C_cor_ values (-20.3±0.8‰ and -20.2±0.6‰, respectively) and they did not differ in δ^13^C_cor_ (Table 4). The highest mean δ^15^N in bison was recorded in the Pašilių (LT) and Mirosławiec (PL) populations (5.6±0.4‰ and 5.1±0.8‰, respectively), and these populations did not differ in δ^15^N (Table 4). The bison population from the Bieszczady Mountains (PL) had the lowest mean δ^13^C_cor_ (-23.6±0.2‰) and the lowest δ^15^N (1.0±0.4‰), this was the only population that significantly differed in isotopic composition from all the other bison populations (Table 4).

**Table 3:** One-way ANOVA for δ^13^C_cor_ and δ^15^N for six European bison *Bison bonasus* and four moose *Alces alces* populations.

Species	Isotope	N	Mean	SD	ANOVA				
					SS	df	MS	*F*	*P* - value
Bison									
	δ^13^C_cor_	73	-21.9	1.9	85.53	5	17.11	66.93	**< 0.0001**
	δ^15^N	73	3.6	1.3	89.01	5	17.80	50.48	**< 0.0001**
Moose									
	δ^13^C_cor_	23	-22.0	0.7	2.50	3	0.83	1.81	0.18
	δ^15^N	23	3.0	1.3	12.68	3	4.23	3.02	0.06

Significant assays are shown in bold. SS-sum of squares; MS-mean square.

**Table 4:** Pairwise divergence between Polish and Lithuanian populations of *Bison bonasus* (N = 73) based on δ^13^C_cor_ and δ^15^N.

Population	δ^13^C_cor_ *F* = 66.93					
	Bieszczady M = -23.56	Knyszyn M = -22.56	Pašilių M = -22.10	Białowieża M = -22.45	Mirosławiec M = -20.26	Borki M = -20.17
Bieszczady		**0.031**	**< 0.001**	**0.011**	**< 0.001**	**< 0.001**
Knyszyn	**0.031**		0.699	0.998	**< 0.001**	**< 0.001**
Pašilių	**< 0.001**	0.699		0.884	**< 0.001**	**< 0.001**
Białowieża	**0.011**	0.998	0.884		**< 0.001**	**< 0.001**
Mirosławiec	**< 0.001**	**< 0.001**	**< 0.001**	**< 0.001**		0.999
Borki	**< 0.001**	**< 0.001**	**< 0.001**	**< 0.001**	0.999	
	δ^15^N *F* = 50.48					
	Bieszczady M = 1.04	Knyszyn M = 4.03	Pašilių M = 5.62	Białowieża M = 2.99	Mirosławiec M = 5.11	Borki M = 4.12
Bieszczady		**< 0.001**	**< 0.001**	**< 0.001**	**< 0.001**	**< 0.001**
Knyszyn	**< 0.001**		**0.001**	**0.011**	**0.006**	0.999
Pašilių	**< 0.001**	**0.001**		**< 0.001**	0.755	**0.002**
Białowieża	**< 0.001**	**0.011**	**< 0.001**		**< 0.001**	**< 0.001**
Mirosławiec	**< 0.001**	**0.006**	0.755	**< 0.001**		**0.016**
Borki	**< 0.001**	0.999	**0.002**	**< 0.001**	**0.016**	

Significant post-hoc assays with *P* < 0.05 (Tukey’s HSD for unequal n) are shown in bold.

There were no significant differences in δ^13^C_cor_ and δ^15^N among moose from the four considered populations (Table 3). In contrast to bison, moose populations strongly overlapped in δ^13^C_cor_ and δ^15^N (Fig 2).

### Comparison of early Holocene and modern populations

**Fig 5:**
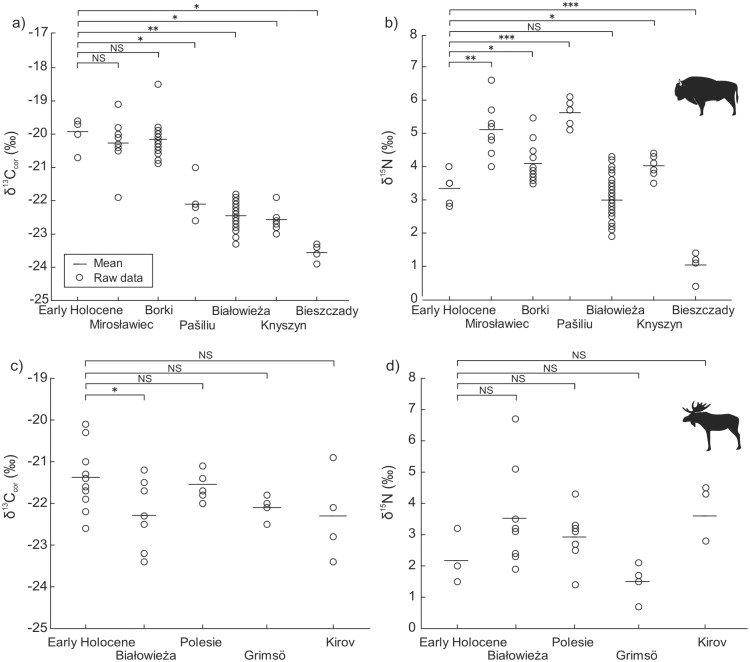
Differences in (a) δ^13^C_cor_ and (b) δ^15^N between early Holocene and modern populations of European bison and (c) (d) moose. One asterisk—*p* = 0.05–0.01, two asterisks—*p* = 0.009–0.001, three asterisks—*p* < 0.001; NS- not significant differences, Mann-Whitney *U*-test for δ^13^C_cor_ and Student’s *t*-test for δ^15^N. Data on carbon and nitrogen stable isotope compositions on early Holocene bison and moose was taken from the literature [16, 47].

Modern bison populations with the exception of Mirosławiec (PL) and Borki (PL) populations in δ^13^C_cor_, and the Białowieża (PL) population in δ^15^N, significantly differed from early Holocene specimens (Fig 5a and 5b). Four populations that differed in δ^13^C_cor_ were characterised by lower values than early Holocene bison (Fig 5a). In contrast, all modern populations of moose had comparable and not significantly different δ^13^C_cor_ and δ^15^N values to the early Holocene specimens (Fig 5c and 5d), with the exception of moose from Białowieża (PL) in δ^13^C_cor_ (Fig 5c).

## Discussion

Modelling showed that only one factor, forest cover, strongly influenced δ^13^C_cor_ in both species. This is consistent with the study concerning the influence of “canopy effect”, including recycling of ^13^C-depleted CO_2_ and the effect of increased shade and humidity of photosynthetic conditions [15, 19], on δ^13^C in plants. Therefore, we interpret changes in δ^13^C as the foraging in forested habitats. Our results indicate that foraging in a dense-canopy forested landscape is the best explanation for carbon isotope variation in interpreting foraging habitat use, therefore, in European conditions can be applied for the reconstruction of foraging habitats of large herbivores in the past. As the mean forest cover in the bison habitats was 10% higher than in the moose habitats, we expected that moose should use open areas (including marshlands, wastelands etc.) to a wider extent [63], as they are not actively confined to forest habitats as bison do, and therefore, have higher δ^13^C_cor_. However, we found similar average δ^13^C_cor_ values in both species. This may be related to the avoidance of agricultural areas by moose [64], which are the most available habitats among the open habitats in the studied area. Because forest cover was the only significant factor affecting δ^13^C_cor_, and explained higher proportion of δ^13^C_cor_ variance in bison, we concluded that this factor was much more important for bison than for moose. It may result from active confinement of bison to forest habitats by managers, and from a stronger effect of canopy shading on the δ^13^C_cor_ values in plants than differences in the diet content due to consumption of different plants or plant organs.

Nitrogen isotope variability in bison was best explained by a model incorporating three factors: forest cover, the average annual temperature, and the use of agricultural crops, among which the first factor had the highest influence. A clear relationship between the importance of forest cover and a decrease in δ^15^N of bison was observed. This relationship may result from higher consumption of browse forage, and consequently lower consumption of ^15^N-enriched grasses and forbs in forested habitats [31]. Thus, nitrogen isotopic composition of bison mirrored different habitat structure and availability of different forage types in the studied populations. In our study δ^15^N of bison was positively correlated with temperature, similarly to what was found in modern populations of red deer in Europe [65] and other studies on plants [22, 45]. The use of agricultural crops by bison increased δ^15^N in their collagen. In many areas bison seasonally visit surrounding agricultural areas and forage on rape (*Brassica napus* L. var. *napus*) and winter cereals like barley (*Hordeum* L.), wheat (*Triticum* L.), rye (*Secale* L.) or hay left by farmers on meadows [59, 66]. They are also supplementary fed with hay or C_4_ maize as in Borki Forest. In a study on red deer (*Cervus elaphus*) diet in Oklahoma (USA), mean δ^15^N in cultivated C_3_ plants was about 3‰ higher than in all the native plants. In the most abundant cultivated crop (winter wheat), δ^15^N was about 1.5‰ higher than the next highest value observed in C_3_ grasses [67]. Studies on soil nitrogen isotope composition confirmed also that soil in undisturbed areas had lower δ^15^N values than in agricultural lands [25], and birds from agricultural landscapes had higher δ^15^N values than those from boreal forest in Canada [68]. In contrast to bison, δ^15^N of moose was not affected by any of the investigated factors.

The range of the δ^13^C_cor_ was greater in bison than in moose, but the δ^15^N isotopic variation was comparable for both species; however, variation in both isotopes resulted from significant inter-population differences in bison, while in moose it mainly resulted from intra-population variation. Isotopic values observed in bison are more homogenous within the populations probably because of feeding on isotopically similar grass/herb dominated patches and as a result of the social behaviour of this herbivore, but also because of winter supplementation, which causes aggregation of bison in large herds and influences spatial organisation in the other seasons [27, 69]. We found a significant positive correlation between δ^13^C_cor_ and δ^15^N in bison, but not in moose. This might indicate that bison populations with higher δ^13^C_cor_ most likely used less-forested habitats, at the same time having higher δ^15^N, which reflects a higher proportion of grassy vegetation (more enriched in δ^15^N than browse vegetation) in their diet.

Because 69% of the free-living bison populations which have been introduced into forest habitats have expanded their range to open areas [28], we expected that bison would select more open areas such as the edges of woodlands, river valleys, glades and clearings when introduced to forest habitats. Such behaviour would yield carbon isotopic signatures characteristic of open habitats, within a range between -21.0‰ and -18.0‰. Surprisingly, bison showed higher than expected inter-population variation in isotopic composition, which could have been influenced by different habitat structure in the surveyed locations. The stable carbon isotope abundance in the bones of bison occurring predominantly in forest habitats (Białowieża, Bieszczady and Knyszyn forests (PL)) ranged from -23.9‰ to -21.8‰, which suggests foraging under the forest canopy. Despite the fact that in the Białowieża Forest, visitation frequency of bison inside forest gaps was almost twice as high as compared to closed, shady forest [70], it was not necessarily reflected in carbon isotopic compositions. It seems that the likelihood of capturing the utilization of small size open environments (glades, river valleys, clearings) in the carbon isotopic composition in large continuous forests is relatively low.

Very high values of δ^13^C_cor_ between -21.9‰ and -18.5‰, in bison from Mirosławiec (PL) and Borki (PL) populations could suggest a substantial foraging in open habitats in contrast to bison from Białowieża (PL) and Bieszczady (PL) populations (lower δ^13^C_cor_). In the case of bison from Mirosławiec (PL) these isotopic results are consistent with the behavior observed in this population that normally utilised a mosaic of open areas and less dense and more exposed Scots pine *Pinus sylvestris* forest patches. In such conditions, δ^13^C values in plants are higher than in plants growing under the dense canopy in much darker deciduous forests [19]. Interestingly, bison populations from Borki and Białowieża (PL), which occupied relatively similar forest habitats (deciduous forest share– 77% and 61%, respectively) [71], differed in their carbon isotope abundance. As expected, high intensity of supplementary feeding with maize of bison from Borki shifted the composition of carbon isotopes towards a value similar to the value expected for feeding in more open habitats. Higher δ^13^C_cor_ and δ^15^N in populations in Mirosławiec (PL) and Pašilių (LT) are probably the result of feeding on meadows and farm crops [27, 57]. As expected, bison from Bieszczady Mountains (PL) had the lowest δ^15^N because of the influence of altitude [46].

In moose, we observed a higher overlap between populations in the isotopic composition of their collagen than in bison. We could interpret this pattern as reflecting large inter-individual differences in habitat use and diet. Utilization of a great variety of habitats [35], as expected, caused higher intra-population variation in δ^13^C_cor_ in moose than in bison. Because none of the considered environmental factors explained δ^15^N in moose, we think that broad values range of this isotope within population might be explained by the diverse diet of moose [35, 38, 39]. Moreover, partial migrations of moose where some individuals migrate and others do not [72], leads to a seasonal shift in habitat and food type selection for some individuals [73, 74]. This behaviour probably increases the variation in δ^13^C_cor_ and δ^15^N between individuals [75]. Similarly to moose, bison may migrate seasonally [59, 76]. However, in contrast to moose, migration did not influence isotopic signature of bison because most of seasonal migrations of the species are inhibited by regular supplementary feeding inside the forests [59, 77]. Compared to gregarious bison, moose are less social and have independent foraging strategies [39, 78]. Therefore, the large variation of carbon and nitrogen isotope values within each population of moose is most probably related to a diverse diet, low sociality of the species and migrations unlimited by management.

Early Holocene European bison differed according to isotopic content from most of modern European bison populations with the exception of Mirosławiec (PL), Borki (PL) (δ^13^C_cor_) and Białowieża (PL) populations (δ^15^N). In Mirosławiec (PL) bison utilize a mosaic of forest patches and open areas (meadows, open military areas, farmland)–environments where the level of the openness may be similar to that occurring in the early Holocene [16, 47, 79]. The δ^13^C values of the bison population in Borki (PL) were similar to those of the Early Holocene bison because of the high share of C_4_ plants in their diet which artificially elevated the δ^13^C_cor_ values to a level similar to that observed in early Holocene bison. Therefore, this isotopic similarity does not correspond to a similar foraging behaviour. Based on stable isotope analysis of early Holocene herbivores from northern Europe, bison was classified as a mixed feeder, whereas moose was classified as a typical browser. Both species were found to have lived in a relatively open tundra-like environment [16]. In the modern moose populations that we studied (with one exception—δ^13^C_cor_ in Białowieża population), the isotopic patterns were similar to those observed in the early Holocene. This confirms a more naturally shaped resource selection by moose related to recolonization of preferred habitats and use of optimal resources as predicted by optimal foraging theory. In contrast, we can see in bison a strong influence of habitat structure and management activities at reintroduction sites, which strongly shapes the pattern of habitat and resource use as reflected in isotopic signatures. Our results support the refugee status of bison that were reintroduced to suboptimal or marginal forest habitats [28], in which management activities have been acting against natural selection pressures. This indicates that a better knowledge of the pre-refugee ecology of species is highly relevant and that this information should serve as a basis in rewilding programmes.

## Conclusions

Our results showed that foraging patterns of bison and moose at large geographical scale are mainly shaped by habitat structure and different history of population recovery—natural recolonization in moose (indicated by more natural selection of resources) *versus* reintroductions supported by extensive management in bison. Influence of forest structure on carbon isotope signatures of studied herbivores supports the “canopy effect” hypothesis. We conclude that subjective selection of areas for species introduction, based on historical (survival of bison only in forest habitats in last centuries) rather than scientific evidence [28, 80], strongly shape foraging patterns of European bison. It also indicates strong dietary plasticity of bison that probably allowed the species to survive replacement of open habitats by forests in the effect of climate change during Pleistocene/Holocene transition [81]. However, survival of bison in forest habitats, that does not offer much food for large grazers or mixed feeders, without supplementary feeding would be difficult, having numerous negative consequences and leading to human-wildlife conflicts [59, 66, 82, 83]. So, introduction of bison to forest habitats and their active management there reinforce the refugee status of bison, act against their natural selection, and lead to unclear, but probably profound physiological and demographic consequences [28]. Thus, the conservation management of European bison should include introductions of bison to more open or mixed habitats that will result in more natural habitat selection by bison and reduce human care.

## Supporting information

S1 Table Values of carbon δ^13^C_coll_ and nitrogen δ^15^N measured in collagen of modern European bison *Bison bonasus* and moose *Alces alces* populations, characteristics of environmental conditions (altitude, mean annual precipitation, mean annual temperature) in analysed populations and farmland utilization by bison. Data on presence/absence (1-yes, 0-no) of farm crop depredation and utilization/lack of utilization (1-yes, 0-no) of maize (C_4_ plant) by bison was taken from the literature [28, 57, 66, 84–86] or interviews with local managers (M. Tracz, personal communication, Western Pomeranian Natural Society, P. Khoyetskyy, personal communication, National Forestry University of Ukraine, Lviv).
(DOC)
