# Supplementary material for: Stable isotope signatures of large herbivore foraging habitats across Europe
Source: PLoS One. 2018 Jan 2;13(1):e0190723. doi: 10.1371/journal.pone.0190723 (PMC5749876; doi:10.1371/journal.pone.0190723)
Supplement: S1 Table — Data on presence/absence (1-yes, 0-no) of farm crop depredation and utilization/lack of utilization (1-yes, 0-no) of maize (C4 plant) by bison was taken from the literature [28, 57, 66, 84–86] or interviews with local managers (M. Tracz, personal communication, Western Pomeranian Natural Society, P. Khoyetskyy, personal communication, National Forestry University of Ukraine, Lviv). (DOC) [file pone.0190723.s001.doc]

**Supporting Information**

**Stable isotope signatures of large herbivore foraging habitats across Europe**

Emilia Hofman-Kamińska, Hervé Bocherens, Tomasz Borowik, Dorothée G. Drucker and Rafał Kowalczyk

Corresponding author: Emilia Hofman-Kamińska, mail: ehofman@ibs.bialowieza.pl

**S1 Table.** **Values of carbon δ^13^C_coll_ and nitrogen δ^15^N measured in collagen of modern European bison *Bison bonasus* and moose *Alces alces* populations, characteristics of environmental conditions (altitude, mean annual precipitation, mean annual temperature) in analyzed populations and farmland utilization by bison.**

| Specimen numbers or ID numbers | Species | Country | Locality | Year of death | %Cc | %Nc | C/N | δ^13^C_coll_  (‰) | ∆^13^C_atm_ | δ ^13^C_cor_  (‰) | δ ^15^N  (‰) | Altitude  (m.a.s.l.) | Mean annual precipitation  (mm) | Mean annual temperature  (°C) | Forest cover  (%) | C4 food | Crop damage | Museum name |
| --- | --- | --- | --- | --- | --- | --- | --- | --- | --- | --- | --- | --- | --- | --- | --- | --- | --- | --- |
| 437 | B. bonasus | Belarus | Pripjatsk | 2011 | 39.9 | 14.3 | 3.3 | -21.6 | 2.15 | -19.4 | 6.4 | 137 | 623 | 7.0 | 69.5 | 1 | 1 | PNP |
| 438 | B. bonasus | Belarus | Pripjatsk | 2011 | 42.4 | 15.2 | 3.3 | -20.8 | 2.15 | -18.7 | 6.4 | 137 | 623 | 7.0 | 69.5 | 1 | 1 | PNP |
| 150 | B. bonasus | Lithuania | Pašilių | 1994 | 40.8 | 14.8 | 3.2 | -22.5 | 1.49 | -21.0 | 5.3 | 61 | 616 | 6.3 | 80.9 | 1 | 1 | DAPLVA |
| 151 | B. bonasus | Lithuania | Pašilių | 1994 | 41.8 | 15.4 | 3.2 | -24.1 | 1.49 | -22.6 | 6.1 | 61 | 616 | 6.3 | 80.9 | 1 | 1 | DAPLVA |
| 152 | B. bonasus | Lithuania | Pašilių | 1994 | 39.4 | 14.4 | 3.2 | -24.1 | 1.49 | -22.6 | 5.9 | 61 | 616 | 6.3 | 80.9 | 1 | 1 | DAPLVA |
| 153 | B. bonasus | Lithuania | Pašilių | 1994 | 39.4 | 14.6 | 3.2 | -23.7 | 1.49 | -22.2 | 5.7 | 61 | 616 | 6.3 | 80.9 | 1 | 1 | DAPLVA |
| 154 | B. bonasus | Lithuania | Pašilių | 1994 | 36.5 | 13.8 | 3.1 | -23.6 | 1.49 | -22.1 | 5.1 | 61 | 616 | 6.3 | 80.9 | 1 | 1 | DAPLVA |
| 1 | B. bonasus | Poland | Białowieża | 2009 | 42.6 | 15.2 | 3.3 | -25.2 | 2.06 | -23.1 | 2.8 | 161 | 585 | 6.9 | 78.5 | 0 | 1 | MRI PAS |
| 169750 | B. bonasus | Poland | Białowieża | 2009 | 42.4 | 15.3 | 3.2 | -24.5 | 2.06 | -22.5 | 2.2 | 158 | 585 | 6.9 | 78.5 | 0 | 1 | MRI PAS |
| 3 | B. bonasus | Poland | Białowieża | 2009 | 42.1 | 15.0 | 3.3 | -24.3 | 2.06 | -22.2 | 3.1 | 162 | 580 | 7.0 | 78.5 | 0 | 0 | MRI PAS |
| 169607 | B. bonasus | Poland | Białowieża | 2007 | 44.4 | 15.5 | 3.3 | -25.0 | 1.97 | -23.1 | 2.1 | 175 | 581 | 6.9 | 78.5 | 0 | 0 | MRI PAS |
| 169601 | B. bonasus | Poland | Białowieża | 2007 | 40.5 | 14.4 | 3.3 | -23.8 | 1.97 | -21.8 | 3.3 | 172 | 581 | 6.9 | 78.5 | 0 | 0 | MRI PAS |
| 169562 | B. bonasus | Poland | Białowieża | 2006 | 43.3 | 15.4 | 3.3 | -24.8 | 1.93 | -22.8 | 3.0 | 161 | 584 | 6.9 | 78.5 | 0 | 0 | MRI PAS |
| 169610 | B. bonasus | Poland | Białowieża | 2007 | 46.4 | 15.4 | 3.5 | -24.5 | 1.97 | -22.6 | 3.4 | 168 | 580 | 7.0 | 78.5 | 0 | 0 | MRI PAS |
| 169628 | B. bonasus | Poland | Białowieża | 2008 | 43.5 | 15.3 | 3.3 | -24.3 | 2.01 | -22.3 | 2.9 | 162 | 582 | 6.9 | 78.5 | 0 | 0 | MRI PAS |
| 169751 | B. bonasus | Poland | Białowieża | 2009 | 42.7 | 14.7 | 3.4 | -24.1 | 2.06 | -22.0 | 4.0 | 159 | 585 | 7.0 | 78.5 | 0 | 1 | MRI PAS |
| 169762 | B. bonasus | Poland | Białowieża | 2009 | 42.3 | 14.5 | 3.4 | -24.0 | 2.06 | -22.0 | 2.2 | 181 | 581 | 6.9 | 78.5 | 0 | 0 | MRI PAS |
| 169801 | B. bonasus | Poland | Białowieża | 2010 | 42.7 | 14.5 | 3.4 | -23.9 | 2.10 | -21.8 | 3.5 | 163 | 572 | 7.0 | 78.5 | 0 | 0 | MRI PAS |
| 169855 | B. bonasus | Poland | Białowieża | 2011 | 42.8 | 14.9 | 3.4 | -24.4 | 2.15 | -22.3 | 2.5 | 163 | 581 | 7.0 | 78.5 | 0 | 0 | MRI PAS |
| 169797 | B. bonasus | Poland | Białowieża | 2007 | 43.0 | 14.5 | 3.4 | -23.9 | 1.97 | -21.9 | 2.5 | 165 | 580 | 7.0 | 78.5 | 0 | 0 | MRI PAS |
| 169647 | B. bonasus | Poland | Białowieża | 2008 | 41.9 | 14.2 | 3.4 | -24.7 | 2.01 | -22.7 | 3.0 | 168 | 580 | 7.0 | 78.5 | 0 | 0 | MRI PAS |
| 164651 | B. bonasus | Poland | Białowieża | 1996 | 43.2 | 14.9 | 3.4 | -24.3 | 1.55 | -22.7 | 2.3 | 168 | 580 | 7.0 | 78.5 | 0 | 0 | MRI PAS |
| 169534 | B. bonasus | Poland | Białowieża | 2006 | 43.5 | 15.1 | 3.4 | -24.6 | 1.93 | -22.6 | 2.3 | 168 | 580 | 7.0 | 78.5 | 0 | 0 | MRI PAS |
| 169599 | B. bonasus | Poland | Białowieża | 2007 | 42.9 | 14.7 | 3.4 | -24.1 | 1.97 | -22.2 | 3.0 | 181 | 575 | 6.9 | 78.5 | 0 | 0 | MRI PAS |
| 169847 | B. bonasus | Poland | Białowieża | 2011 | 39.4 | 13.5 | 3.4 | -24.9 | 2.15 | -22.8 | 3.9 | 181 | 575 | 6.9 | 78.5 | 0 | 0 | MRI PAS |
| 169760 | B. bonasus | Poland | Białowieża | 2009 | 38.8 | 13.5 | 3.4 | -24.1 | 2.06 | -22.0 | 4.2 | 163 | 584 | 7.0 | 78.5 | 0 | 0 | MRI PAS |
| 168760 | B. bonasus | Poland | Białowieża | 2003 | 39.8 | 13.4 | 3.5 | -24.3 | 1.81 | -22.5 | 2.2 | 144 | 581 | 7.0 | 78.5 | 0 | 1 | MRI PAS |
| 169800 | B. bonasus | Poland | Białowieża | 2010 | 41.5 | 14.2 | 3.4 | -24.7 | 2.10 | -22.6 | 2.6 | 181 | 581 | 6.8 | 78.5 | 0 | 1 | MRI PAS |
| 169412 | B. bonasus | Poland | Białowieża | 2004 | 40.1 | 13.8 | 3.4 | -23.9 | 1.85 | -22.0 | 3.0 | 181 | 581 | 6.8 | 78.5 | 0 | 1 | MRI PAS |
| 166933 | B. bonasus | Poland | Białowieża | 1998 | 40.7 | 13.9 | 3.4 | -24.4 | 1.62 | -22.8 | 1.9 | 181 | 581 | 6.8 | 78.5 | 0 | 1 | MRI PAS |
| 169792 | B. bonasus | Poland | Białowieża | 2010 | 42.2 | 14.4 | 3.4 | -24.5 | 2.10 | -22.4 | 3.4 | 180 | 574 | 7.0 | 78.5 | 0 | 0 | MRI PAS |
| 169455 | B. bonasus | Poland | Białowieża | 2005 | 41.8 | 14.6 | 3.3 | -24.8 | 1.89 | -22.9 | 2.5 | 181 | 581 | 6.8 | 78.5 | 0 | 1 | MRI PAS |
| 163670 | B. bonasus | Poland | Białowieża | 1994 | 42.4 | 14.6 | 3.4 | -23.8 | 1.49 | -22.3 | 3.8 | 181 | 581 | 6.8 | 78.5 | 0 | 1 | MRI PAS |
| 169456 | B. bonasus | Poland | Białowieża | 2005 | 42.9 | 14.7 | 3.4 | -24.0 | 1.89 | -22.1 | 3.1 | 163 | 584 | 7.0 | 78.5 | 0 | 0 | MRI PAS |
| 169863 | B. bonasus | Poland | Białowieża | 2011 | 42.2 | 14.5 | 3.4 | -24.7 | 2.15 | -22.5 | 4.3 | 144 | 581 | 7.0 | 78.5 | 0 | 1 | MRI PAS |
| 169629 | B. bonasus | Poland | Białowieża | 2008 | 42.8 | 15.1 | 3.3 | -24.8 | 2.01 | -22.8 | 3.1 | 162 | 582 | 6.9 | 78.5 | 0 | 0 | MRI PAS |
| 169415 | B. bonasus | Poland | Białowieża | 2004 | 41.3 | 15.0 | 3.2 | -25.1 | 1.85 | -23.3 | 3.3 | 168 | 580 | 7.0 | 78.5 | 0 | 0 | MRI PAS |
| 169202 | B. bonasus | Poland | Białowieża | 2004 | 44.3 | 15.7 | 3.3 | -24.0 | 1.85 | -22.2 | 3.2 | 180 | 574 | 7.0 | 78.5 | 0 | 0 | MRI PAS |
| 168723 | B. bonasus | Poland | Białowieża | 2003 | 44.0 | 15.7 | 3.3 | -23.6 | 1.81 | -21.8 | 2.3 | 152 | 582 | 6.9 | 78.5 | 0 | 0 | MRI PAS |
| 170223 | B. bonasus | Poland | Białowieża | 2013 | 41.8 | 14.8 | 3.3 | -24.5 | 2.24 | -22.3 | 3.5 | 177 | 582 | 6.9 | 78.5 | 0 | 0 | MRI PAS |
| 170249 | B. bonasus | Poland | Białowieża | 2013 | 43.4 | 15.6 | 3.3 | -24.9 | 2.24 | -22.7 | 2.7 | 173 | 581 | 7.0 | 78.5 | 0 | 0 | MRI PAS |
| 169886 | B. bonasus | Poland | Białowieża | 2012 | 41.5 | 14.8 | 3.3 | -25.3 | 2.20 | -23.1 | 3.6 | 176 | 581 | 6.9 | 78.5 | 0 | 0 | MRI PAS |
| 59 | B. bonasus | Poland | Bieszczady | 2004 | 41.5 | 14.2 | 3.4 | -25.3 | 1.85 | -23.4 | 1.4 | 1125 | 937 | 3.6 | 86.9 | 0 | 0 | CWRS |
| 60 | B. bonasus | Poland | Bieszczady | 2004 | 40.6 | 14.4 | 3.3 | -25.1 | 1.85 | -23.3 | 1.1 | 689 | 819 | 5.2 | 79.1 | 0 | 0 | CWRS |
| 61 | B. bonasus | Poland | Bieszczady | 2009 | 40.5 | 14.5 | 3.3 | -25.9 | 2.06 | -23.9 | 1.1 | 623 | 794 | 5.4 | 79.1 | 0 | 0 | CWRS |
| 62 | B. bonasus | Poland | Bieszczady | 2011 | 40.9 | 14.3 | 3.3 | -25.8 | 2.15 | -23.6 | 0.4 | 711 | 822 | 5.0 | 79.1 | 0 | 0 | CWRS |
| 88 | B. bonasus | Poland | Bieszczady | 2011 | 39.8 | 14.3 | 3.3 | -25.7 | 2.15 | -23.6 | 1.2 | 702 | 819 | 5.1 | 79.1 | 0 | 0 | CWRS |
| 253 | B. bonasus | Poland | Borki | 2010 | 41.5 | 14.3 | 3.4 | -22.1 | 2.10 | -20.0 | 3.6 | 176 | 660 | 6.4 | 46.3 | 1 | 0 | BFD |
| 254 | B. bonasus | Poland | Borki | 2010 | 40.8 | 14.3 | 3.3 | -21.9 | 2.10 | -19.8 | 4.3 | 176 | 660 | 6.4 | 46.3 | 1 | 0 | BFD |
| 255 | B. bonasus | Poland | Borki | 2010 | 40.1 | 13.6 | 3.4 | -22.9 | 2.10 | -20.8 | 4.9 | 176 | 660 | 6.4 | 46.3 | 1 | 0 | BFD |
| 260 | B. bonasus | Poland | Borki | 2010 | 42.1 | 15.2 | 3.2 | -20.6 | 2.10 | -18.5 | 4.5 | 176 | 660 | 6.4 | 46.3 | 1 | 0 | BFD |
| 261 | B. bonasus | Poland | Borki | 2010 | 40.5 | 14.5 | 3.3 | -23.0 | 2.10 | -20.9 | 4.0 | 176 | 660 | 6.4 | 46.3 | 1 | 0 | BFD |
| 408 | B. bonasus | Poland | Borki | 2011 | 44.8 | 16.4 | 3.2 | -22.7 | 2.15 | -20.5 | 3.7 | 176 | 660 | 6.4 | 46.3 | 1 | 0 | BFD |
| 409 | B. bonasus | Poland | Borki | 2011 | 46.8 | 16.5 | 3.3 | -22.4 | 2.15 | -20.2 | 3.8 | 176 | 660 | 6.4 | 46.3 | 1 | 0 | BFD |
| 410 | B. bonasus | Poland | Borki | 2011 | 44.0 | 15.9 | 3.2 | -22.0 | 2.15 | -19.9 | 3.9 | 176 | 660 | 6.4 | 46.3 | 1 | 0 | BFD |
| 411 | B. bonasus | Poland | Borki | 2011 | 43.8 | 15.9 | 3.2 | -22.5 | 2.15 | -20.3 | 3.9 | 176 | 660 | 6.4 | 46.3 | 1 | 0 | BFD |
| 412 | B. bonasus | Poland | Borki | 2011 | 44.1 | 15.6 | 3.3 | -22.8 | 2.15 | -20.6 | 3.5 | 176 | 660 | 6.4 | 46.3 | 1 | 0 | BFD |
| 413 | B. bonasus | Poland | Borki | 2011 | 44.6 | 16.1 | 3.2 | -22.6 | 2.15 | -20.4 | 5.5 | 176 | 660 | 6.4 | 46.3 | 1 | 0 | BFD |
| 414 | B. bonasus | Poland | Borki | 2011 | 44.5 | 16.0 | 3.3 | -22.2 | 2.15 | -20.1 | 3.8 | 176 | 660 | 6.4 | 46.3 | 1 | 0 | BFD |
| 419 | B. bonasus | Poland | Drawsko | 2011 | 41.7 | 15.2 | 3.2 | -24.4 | 2.15 | -22.2 | 4.6 | 105 | 612 | 7.9 | 66.3 | 1 | 1 | WPNS |
| 7 | B. bonasus | Poland | Knyszyn | 2009 | 43.0 | 15.4 | 3.3 | -25.1 | 2.06 | -23.0 | 4.4 | 154 | 580 | 6.7 | 51.6 | 1 | 1 | KFD |
| 8 | B. bonasus | Poland | Knyszyn | 2009 | 44.3 | 15.2 | 3.4 | -24.8 | 2.06 | -22.7 | 3.8 | 164 | 584 | 6.6 | 51.6 | 1 | 1 | KFD |
| 9 | B. bonasus | Poland | Knyszyn | 2009 | 44.5 | 15.5 | 3.4 | -23.9 | 2.06 | -21.9 | 3.5 | 170 | 585 | 6.6 | 51.6 | 1 | 1 | KFD |
| 10 | B. bonasus | Poland | Knyszyn | 2011 | 43.3 | 15.5 | 3.3 | -24.7 | 2.15 | -22.5 | 4.1 | 189 | 589 | 6.4 | 51.6 | 1 | 1 | KFD |
| 11 | B. bonasus | Poland | Knyszyn | 2010 | 41.9 | 15.2 | 3.2 | -24.6 | 2.10 | -22.5 | 4.1 | 162 | 585 | 6.7 | 51.6 | 1 | 1 | KFD |
| 12 | B. bonasus | Poland | Knyszyn | 2010 | 43.5 | 15.6 | 3.3 | -24.7 | 2.10 | -22.6 | 4.1 | 153 | 584 | 6.7 | 51.6 | 1 | 1 | KFD |
| 91 | B. bonasus | Poland | Knyszyn | 2011 | 44.2 | 15.8 | 3.3 | -25.0 | 2.15 | -22.8 | 4.3 | 203 | 594 | 6.4 | 51.6 | 1 | 1 | KFD |
| 92 | B. bonasus | Poland | Knyszyn | 2011 | 41.7 | 15.1 | 3.2 | -24.7 | 2.15 | -22.5 | 3.9 | 183 | 589 | 6.5 | 51.6 | 1 | 1 | KFD |
| 13 | B. bonasus | Poland | Mirosławiec | 2011 | 42.8 | 15.2 | 3.3 | -22.3 | 2.15 | -20.1 | 4.8 | 156 | 628 | 7.5 | 53.8 | 1 | 1 | WPNS |
| 14 | B. bonasus | Poland | Mirosławiec | 2011 | 43.0 | 15.1 | 3.3 | -21.9 | 2.15 | -19.8 | 4.0 | 151 | 631 | 7.5 | 53.8 | 1 | 1 | WPNS |
| 15 | B. bonasus | Poland | Mirosławiec | 2010 | 43.3 | 15.4 | 3.3 | -24.0 | 2.10 | -21.9 | 5.7 | 113 | 616 | 7.8 | 53.8 | 1 | 1 | WPNS |
| 16 | B. bonasus | Poland | Mirosławiec | 2010 | 44.2 | 15.5 | 3.3 | -22.1 | 2.10 | -20.0 | 4.4 | 155 | 625 | 7.5 | 53.8 | 1 | 1 | WPNS |
| 17 | B. bonasus | Poland | Mirosławiec | 2010 | 45.5 | 16.1 | 3.3 | -22.4 | 2.10 | -20.3 | 5.3 | 123 | 616 | 7.7 | 53.8 | 1 | 1 | WPNS |
| 18 | B. bonasus | Poland | Mirosławiec | 2010 | 44.3 | 15.6 | 3.3 | -22.5 | 2.10 | -20.4 | 5.2 | 141 | 621 | 7.6 | 53.8 | 1 | 1 | WPNS |
| 20 | B. bonasus | Poland | Mirosławiec | 2010 | 43.3 | 15.2 | 3.3 | -22.6 | 2.10 | -20.5 | 4.9 | 141 | 621 | 7.6 | 53.8 | 1 | 1 | WPNS |
| 420 | B. bonasus | Poland | Mirosławiec | 2011 | 44.7 | 16.3 | 3.2 | -21.3 | 2.15 | -19.1 | 6.6 | 126 | 610 | 7.6 | 53.8 | 1 | 1 | WPNS |
| 358 | B. bonasus | Ukraine | Danivska | 1990 | 42.8 | 15.3 | 3.3 | -21.9 | 1.36 | -20.6 | 3.3 | 124 | 590 | 7.7 | 63.7 | 1 | 1 | SIZ NAS |
| 361 | B. bonasus | Ukraine | Danivska | 1990 | 40.4 | 14.7 | 3.2 | -22.6 | 1.36 | -21.2 | 4.5 | 124 | 590 | 7.7 | 63.7 | 1 | 1 | SIZ NAS |
| 365 | B. bonasus | Ukraine | Tsumanska | 2000 | 41.9 | 14.9 | 3.3 | -24.5 | 1.69 | -22.8 | 4.1 | 211 | 569 | 7.1 | 45.3 | 1 | 1 | UFNU |
| 55 | A. alces | Belarus | Berezina | 1976 | 43.9 | 14.3 | 3.6 | -23.3 | 1.01 | -22.3 | 3.8 | 180 | 648 | 5.8 | 70.6 | - | - | NPBP |
| 56 | A. alces | Belarus | Berezina | 1974 | 40.3 | 13.7 | 3.4 | -22.2 | 0.96 | -21.3 | 6.5 | 180 | 648 | 5.8 | 70.6 | - | - | NPBP |
| 57 | A. alces | Belarus | Berezina | 1973 | 39.4 | 12.8 | 3.6 | -23.3 | 0.94 | -22.3 | 4.2 | 180 | 648 | 5.8 | 70.6 | - | - | NPBP |
| 58 | A. alces | Belarus | Scharkov | 2011 | 41.1 | 14.1 | 3.4 | -22.7 | 2.15 | -20.6 | 5.1 | 144 | 629 | 5.8 | 20.8 | - | - | NPBP |
| 157 | A. alces | Lithuania | Anyksciai | 2004 | 39.9 | 14.6 | 3.2 | -23.3 | 1.85 | -21.4 | 3.0 | 97 | 615 | 6.1 | 33.0 | - | - | LIH |
| 155 | A. alces | Lithuania | Kretinga | 2008 | 34.4 | 13.3 | 3 | -24.0 | 2.01 | -22.0 | 5.8 | 40 | 758 | 6.8 | 22.1 | - | - | LIH |
| 311 | A. alces | Lithuania | Kursiu | 2009 | 41.9 | 14.8 | 3.3 | -23.3 | 2.06 | -21.3 | 1.4 | 9 | 731 | 7.3 | 61.2 | - | - | LIH |
| 312 | A. alces | Poland | Białowieża | 2012 | 40.3 | 14.2 | 3.3 | -24.7 | 2.20 | -22.5 | 3.5 | 183 | 582 | 6.9 | 78.5 | - | - | MRI PAS |
| 321 | A. alces | Poland | Białowieża | 1995 | 42.5 | 15.3 | 3.2 | -24.7 | 1.52 | -23.2 | 2.3 | 166 | 580 | 7.0 | 78.5 | - | - | MRI PAS |
| 322 | A. alces | Poland | Białowieża | 1995 | 42.8 | 15.9 | 3.1 | -24.0 | 1.52 | -22.5 | 1.9 | 166 | 580 | 7.0 | 78.5 | - | - | MRI PAS |
| 323 | A. alces | Poland | Białowieża | 1995 | 43.3 | 15.7 | 3.2 | -22.8 | 1.52 | -21.2 | 3.1 | 166 | 580 | 7.0 | 78.5 | - | - | MRI PAS |
| 324 | A. alces | Poland | Białowieża | 1995 | 42.5 | 15.6 | 3.2 | -23.0 | 1.52 | -21.5 | 6.7 | 166 | 580 | 7.0 | 78.5 | - | - | MRI PAS |
| 325 | A. alces | Poland | Białowieża | 1995 | 43.7 | 16.0 | 3.2 | -24.9 | 1.52 | -23.4 | 2.4 | 166 | 580 | 7.0 | 78.5 | - | - | MRI PAS |
| 326 | A. alces | Poland | Białowieża | 1995 | 40.8 | 15.0 | 3.2 | -23.2 | 1.52 | -21.7 | 5.1 | 166 | 580 | 7.0 | 78.5 | - | - | MRI PAS |
| 327 | A. alces | Poland | Białowieża | 1995 | 43.8 | 16.1 | 3.2 | -23.9 | 1.52 | -22.3 | 3.2 | 166 | 580 | 7.0 | 78.5 | - | - | MRI PAS |
| 34 | A. alces | Poland | Augustów | 2010 | 43.9 | 14.7 | 3.5 | -24.5 | 2.10 | -22.4 | 0.4 | 142 | 584 | 6.4 | 84.2 | - | - | UB |
| 36 | A. alces | Poland | Augustów | 2010 | 41.2 | 14.3 | 3.4 | -24.7 | 2.10 | -22.6 | 2.9 | 143 | 583 | 6.4 | 84.2 | - | - | UB |
| 35 | A. alces | Poland | Biebrza | 2010 | 42.7 | 14.4 | 3.5 | -24.2 | 2.10 | -22.1 | 4.1 | 128 | 585 | 6.7 | 41.2 | - | - | UB |
| 37 | A. alces | Poland | Biebrza | 2010 | 42.6 | 14.2 | 3.5 | -22.9 | 2.10 | -20.8 | 4.0 | 122 | 568 | 6.8 | 41.2 | - | - | UB |
| 415 | A. alces | Poland | Borki | 2011 | 43.7 | 15.8 | 3.2 | -23.5 | 2.15 | -21.4 | 2.4 | 176 | 660 | 6.4 | 46.3 | - | - | BNP |
| 38 | A. alces | Poland | Mazury | 2010 | 40.5 | 13.3 | 3.5 | -24.0 | 2.10 | -21.9 | 2.1 | 87 | 690 | 6.8 | 20.4 | - | - | UB |
| 337 | A. alces | Poland | Polesie | 2012 | 42.9 | 15.9 | 3.1 | -24.2 | 2.20 | -22.0 | 4.3 | 191 | 519 | 7.4 | 46.5 | - | - | PNP PL |
| 338 | A. alces | Poland | Polesie | 2013 | 42.4 | 15.9 | 3.1 | -23.3 | 2.24 | -21.1 | 2.7 | 191 | 519 | 7.4 | 46.5 | - | - | PNP PL |
| 339 | A. alces | Poland | Polesie | 2013 | 44.6 | 16.3 | 3.2 | -23.9 | 2.24 | -21.7 | 3.1 | 191 | 519 | 7.4 | 46.5 | - | - | PNP PL |
| 340 | A. alces | Poland | Polesie | 2012 | 42.5 | 15.7 | 3.2 | -23.3 | 2.20 | -21.1 | 2.5 | 191 | 519 | 7.4 | 46.5 | - | - | PNP PL |
| 416 | A. alces | Poland | Polesie | 2010 | 43.6 | 15.6 | 3.3 | -23.9 | 2.10 | -21.8 | 3.3 | 191 | 519 | 7.4 | 46.5 | - | - | PNP PL |
| 417 | A. alces | Poland | Polesie | 2010 | 43.0 | 15.6 | 3.2 | -23.8 | 2.10 | -21.7 | 3.2 | 191 | 519 | 7.4 | 46.5 | - | - | PNP PL |
| 418 | A. alces | Poland | Polesie | 2013 | 44.3 | 16.1 | 3.2 | -23.6 | 2.24 | -21.4 | 1.4 | 191 | 519 | 7.4 | 46.5 | - | - | PNP PL |
| 29 | A. alces | Russia | Kirov | 2010 | 42.8 | 14.2 | 3.5 | -24.9 | 2.10 | -22.8 | 2.8 | 171 | 623 | 2.3 | 80.4 | - | - | ZRRIGMFF |
| 30 | A. alces | Russia | Kirov | 2009 | 44.7 | 15.5 | 3.4 | -23.0 | 2.06 | -20.9 | 4.5 | 171 | 623 | 2.3 | 80.4 | - | - | ZRRIGMFF |
| 32 | A. alces | Russia | Kirov | 2009 | 43.1 | 15.0 | 3.4 | -25.4 | 2.06 | -23.4 | 4.3 | 171 | 623 | 2.3 | 80.4 | - | - | ZRRIGMFF |
| 33 | A. alces | Russia | Kirov | 2010 | 44.4 | 14.6 | 3.5 | -24.2 | 2.10 | -22.1 | 2.8 | 171 | 623 | 2.3 | 80.4 | - | - | ZRRIGMFF |
| 31 | A. alces | Russia | Slobodskoj | 2010 | 44.7 | 15.3 | 3.4 | -23.1 | 2.10 | -21.0 | 1.7 | 174 | 635 | 2.4 | 56.6 | - | - | ZRRIGMFF |
| A 13-2007 | A. alces | Sweden | Grimsö | 2007 | 43.2 | 15.4 | 3.3 | -24.0 | 1.97 | -22.0 | 1.7 | 114 | 663 | 5.4 | 86.8 | - | - | GWRS |
| A 7-2010 | A. alces | Sweden | Grimsö | 2010 | 42.0 | 15.0 | 3.3 | -23.9 | 2.10 | -21.8 | 1.5 | 114 | 663 | 5.4 | 86.8 | - | - | GWRS |
| GE 24-12 | A. alces | Sweden | Grimsö | 2012 | 43.6 | 15.4 | 3.3 | -24.7 | 2.20 | -22.5 | 2.1 | 114 | 663 | 5.4 | 86.8 | - | - | GWRS |
| A6-2013 | A. alces | Sweden | Grimsö | 2013 | 43.4 | 15.5 | 3.3 | -24.3 | 2.24 | -22.1 | 0.7 | 114 | 663 | 5.4 | 86.8 | - | - | GWRS |

Data on presence/absence (1-yes, 0-no) of farm crop depredation and utilization/lack of utilization (1-yes, 0-no) of maize (C_4_ plant) by bison was taken from the literature [1-6] or interviews with local managers (M. Tracz, personal communication, Western Pomeranian Natural Society, P. Khoyetskyy, personal communication, National Forestry University of Ukraine, Lviv).

%Cc and %Nc -the weight percent of carbon and nitrogen in the collagen final product, C/N- carbon to nitrogen atomic ratios in measured samples, δ13Ccoll -values of carbon isotopes measured in the collagen, δ13Ccor- values of δ13Ccoll corrected for the value of ∆13Catm.

MRIPAS - Mammal Research Institute PAS, BFD- Borki Forest District, WPNS - Western Pomeranian Nature Society, CWRS - Carpathian Wildlife Research Station, KFD - Knyszyn Forest District, DAPLVA - Department of Anatomy and Physiology of Lithuanian Veterinary Academy, SIZ NAS - Schmalhausen Institute of Zoology, NAS, UFNU - Ukrainian National Forestry University, PNP - Pripjatskijj National Park, PNP PL - Poleski National Park, Poland, BNP - Biebrza National Park, LIH - Lithuanian Institute of History, NPBP - National Park, "Belovezhskaya Pushcha", GWRS - Grimsö Wildlife Research Station, ZRRIGMFF - Zhitkov Russian Research Institute of Game Management and Fur Farming, UB - University of Białymstok.

The study was based on museum specimens. No animals were killed specifically for this study. No permits were required for the described study. Specimens with collection numbers are accessible by others in a permanent repository.

References

1. Hofman-Kamińska E, Kowalczyk R. Farm Crops Depredation by European Bison (*Bison bonasus*) in the Vicinity of Forest Habitats in Northeastern Poland. Environ Manage. 2012;50(4):530-41. doi: 10.1007/s00267-012-9913-7. PubMed PMID: ISI:000308652800003.

2. Kerley GIH, Kowalczyk R, Cromsigt JPGM. Conservation implications of the refugee species concept and the European bison: king of the forest or refugee in a marginal habitat? Ecography. 2012;35(6):519-29. doi: 10.1111/j.1600-0587.2011.07146.x. PubMed PMID: ISI:000304765800005.

3. Kozlo PG. Analysis of reintroduction and formation of European bison populations in Belarus. European Bison Conservation Newsletter. 2013;6:21-36.

4. Olech W, Perzanowski K. Best practices manual for protection of European Bison. Warsaw: Coordination Center for Environmental Projects; 2014. 96 p.

5. Balčiauskas L. European bison (*Bison bonasus*) in Lithuania: status and possibilities of range extension. Acta Zoologica Lituanica. 1999;9(3):3-18. doi: 10.1080/13921657.1999.10512295.

6. Wyrobek K, Żoch K. Żubry w Puszczy Boreckiej (European bison in Borecka Forest). European Bison Conservation Newsletter. 2011;4:95-100.
